# Small-Molecule Loaded Biomimetic Biphasic Scaffold for Osteochondral Regeneration: An In Vitro and In Vivo Study

**DOI:** 10.3390/bioengineering10070847

**Published:** 2023-07-17

**Authors:** Chih-Hsiang Fang, Yi-Wen Lin, Chung-Kai Sun, Jui-Sheng Sun

**Affiliations:** 1 Trauma and Emergency Center, China Medical University Hospital, No. 2, Xueshi Road, North Dist., Taichung City 40447, Taiwan; danny07291991@hotmail.com; 2 Institute of Biomedical Engineering, College of Medicine, National Taiwan University, No. 1, Sec. 4, Roosevelt Road, Taipei 10617, Taiwan; 3 Institute of Traditional Medicine, School of Medicine, National Yang Ming Chiao Tung University, No. 155, Sec. 2, Linong Street, Taipei 11221, Taiwan; 4 Department of Orthopedic Surgery, En Chu Kong Hospital, No. 399, Fuxing Road, New Taipei City 23741, Taiwan; 5 Department of Orthopedic Surgery, National Taiwan University Hospital, No. 7, Chung-Shan South Road, Taipei 10002, Taiwan

**Keywords:** metformin, kartogenin, cartilage, bone, tissue engineering

## Abstract

Osteoarthritis is a prevalent musculoskeletal disorder in the elderly, which leads to high rates of morbidity. Mesenchymal stem cells (MSCs) are a promising approach to promote tissue regeneration in the absence of effective long-term treatments. Small molecules are relatively inexpensive and can selectively alter stem cell behavior during their differentiation, making them an attractive option for clinical applications. In this study, we developed an extracellular matrix (ECM)-based biphasic scaffold (BPS) loaded with two small-molecule drugs, kartogenin (KGN) and metformin (MET). This cell-free biomimetic biphasic scaffold consists of a bone (gelatin/hydroxyapatite scaffold embedded with metformin [GHSM]) and cartilage (nano-gelatin fiber embedded with kartogenin [NGFK]) layer designed to stimulate osteochondral regeneration. Extracellular matrix (ECM)-based biomimetic scaffolds can promote native cell recruitment, infiltration, and differentiation even in the absence of additional growth factors. The biphasic scaffold (BPS) showed excellent biocompatibility in vitro, with mesenchymal stem cells (MSCs) adhering, proliferating, and differentiated on the biomimetic biphasic scaffolds (GHSM and NGFK layers). The biphasic scaffolds upregulated both osteogenic and chondrogenic gene expression, sulfated glycosaminoglycan (sGAG), osteo- and chondrogenic biomarker, and relative mRNA gene expression. In an in vivo rat model, histo-morphological staining showed effective regeneration of osteochondral defects. This novel BPS has the potential to enhance both subchondral bone repair and cartilage regeneration, demonstrating excellent effects on cell homing and the recruitment of endogenous stem cells.

## 1. Introduction

Due to the lack of blood vessels and perichondrium, the articular surface has limited self-healing ability [1] and a poor capacity to repair [2]. Consequently, full-thickness articular defects common in young and active individuals can lead to cartilage degeneration, osteochondral damage, and eventually to osteoarthritis (OA) [3]. OA is a significant cause of mobility problems and reduced quality of life among the elderly [4].

Recent studies have focused on reconstructing hyaline cartilage in osteoarthritis. Surgical strategies for osteochondral reconstruction have been extensively studied to restore joint architecture and activity [5]. Several methods for treating full-thickness articular damage, such as microfracture [6], mosaicplasty [7], and autologous chondrocytes implantation [8] have been attempted. However, these procedures have limitations, such as a limited number of donor sites, the shape and size of autologous grafts, and in vitro dedifferentiation of chondrocytes during the passage [9]. In studies of osteochondral regeneration, mesenchymal stem cells (MSCs) may overcome many of the limitations of past procedures [10]. However, the traditional method of transplanting cell suspensions failed to rescue osteochondral defects in large animals, as MSCs did not settle and survive when directly administered at the defect site [11]. To ensure that these cells can attach to the sites of defects, a more effective method to deliver MSCs is needed. Recent advances in functional tissue engineering have demonstrated that in addition to facilitating stem cell seeding and adhesion for osteochondral implantation [12], collagen and hyaluronic acid in scaffolds can also promote cell proliferation and cartilage differentiation [13]. Despite recent investigations into scaffolded or scaffold-free systems for cartilage regeneration [14], achieving sufficient thickness in the repair of osteochondral defects remains challenging.

The correct choice of biomaterials, scaffold design, and fabrication method is essential for the development of suitable scaffolds. Among the interstitial extracellular matrices (ECMs) in animals, collagen is the most abundant structural material [15]. However, exogenous collagen can induce in vivo immunogenic and antigenic responses [16]. Derived from the incomplete hydrolysis of collagen, with low levels of phenylalanine and without tyrosine and tryptophan, gelatin forms fewer aromatic radicals and has a decreased antigenic response in vivo [17]. With its lower cost and less immunogenicity, gelatin is considered a superior degradable biomaterial for medical, pharmaceutical, and scaffold applications in tissue engineering [17]. The use of nanofibers is required to accurately recreate the tissue alignment and nanoscale structures in scaffolds. The nanofibers’ scaffold production is recommended in modern tissue engineering, as it offers the possibility to control the surface architecture of a higher compliance fiber to promote contact guidance effects on stem cells proliferation [18].

Metformin (1,1-dimethylbiguanide, MET) lessens hyperglycemia and insulin resistance and is one of the first-line therapies for type II diabetes. Metformin has also shown promising cardiovascular benefits [19] as well as anticancer/neuroprotective [20] and anti-inflammatory [21] effects and also improved bone metabolism [22]. Metformin (MET) also has a favorable safety profile [23]. Kartogenin can promote chondrocytes differentiation both in vitro and in vivo. Kartogenin (KGN) stimulates MSCs to differentiate into matrix-producing chondrocytes via increasing the expression of type II collagen (Col2a1), aggrecan (Acan), the sex determining region Y (SRY)-box 9 (Sox9), and tissue inhibitors of metalloproteinase (TIMPs); it induces chondrogenesis by up-regulation of the Runt-related transcription factor 1 (Runx1) transcription [24] and also controls Runx2 downstream genes, which prevents chondrocyte hypertrophy [25].

Similar to the multiscale and complex organization of the natural extracellular matrix, the strategy for the development of an osteochondral regeneration is also extremely complicated. When the fabrication of osteochondral (OC) tissue scaffold is bio-mimic to the physiological properties of natural tissue, it will enhance the efficacy of osteochondral (OC) defects repair. One of the critical aspects in osteochondral (OC) defects repair is that there must be an efficient interface between both osteo and chondral tissues [26]. Osteochondral scaffolds must have a three-dimensional network of interconnected pores to allow cell growth, nutrient transport, and metabolic waste removal. In addition, scaffolds must degrade and resorb at a controlled rate while allowing cells seeded into the 3D construct to attach, spread, and proliferate [27]. The subchondral matrix should mimic cancellous bone which has sufficient mechanical strength to withstand compressive loads and can support softer materials used for articular cartilage regeneration. In this study, we aim to design and apply a cell-free, small-molecule loaded biomimetic scaffold that enables osteochondral defect regeneration. The scaffold has synergism of metformin (MET) and kartogenin (KGN) molecules, which could induce the autologous source MSCs differentiation and promote cartilage and subchondral bone regeneration.

## 2. Materials and Methods

The experimental design and flow chart of this study was presented as Figure S1.

### 2.1. Materials

Sodium hydroxide (NaOH, CAS# 1310-73-2) was obtained from Showa-Denko (Tokyo, Japan). Orthophosphoric acid (H_3_PO_4_, CAS# 7664-38-2) was obtained from J.T. Baker (Center Valley, Radnor, PA, USA). Hydrochloric acid (HCl, CAS# 7647-01-0), calcium hydroxide (Ca(OH)_2_, CAS# 1305-62-0), glutaraldehyde (GA, CAS# 111-30-8), kartogenin (KGN; CAS# 4727-31-5), metformin (MET; CAS# 1115-70-4), and gelatin (CAS# 9000-70-8) were purchased from Sigma-Aldrich (St. Louis, MO, USA).

### 2.2. Preparation of Nano-Gelatin Fiber Embedded with Kartogenin (NGFK)

A homogeneous solution of 10% gelatin and 10 M kartogenin (KGN) was prepared at 50 °C through a magnetic stirring for 30 min. The temperature was then maintained between 45 and 70 °C using an internal heater (AREX-6; VELP Scientifica, Deer Park, NY, USA) as the solution was transferred to a 10 mL syringe (with an inner diameter of needle outlet of 1.5 mm). The solution was injected through a needle with a 20 kV potential and was applied to droplets after being connected to a high-voltage power supply (SC-PME50; Cosmi Global Co. Ltd., New Taipei City, Taiwan). A distance of 7 cm was set between the collector and capillary tip, and aluminum foil was used to cover the collector. To ensure complete solvent removal, the mats were left for at least 6 h. After 24 h of glutaraldehyde vapor crosslinking (Sigma-Aldrich), the fiber samples were rinsed in ethanol (20 times) before being dried.

### 2.3. Preparation of Metformin Embedded Gelatin/Hydroxyapatite Scaffold (GHSM)

In situ for mineralization of hydroxyapatite (Hap) on gelatin fibrils was achieved by co-precipitating orthophosphoric acid and calcium hydroxide. To obtain a 1:1 weight ratio of gelatin/Hap, 3.665 g of Ca(OH)_2_ was added to 95 mL 3.7 wt% gelatin solution and allowed to completely dissolve. A 0.15 M solution of H_3_PO_4_ (1.95 mL, at 85 °C) was added drop-wise at a rate of 0.3 mL/min, followed by continuous magnetic stirring for 24 h. The pH was adjusted to 9 to facilitate pure nano-HAp precipitation, and then further adjusted to a neutral equivalent. Metformin (MET) was added to achieve a final concentration of 50 μM. Finally, 0.1% microbial transglutaminase (mTGase; Activa, Ajinomoto, Japan) was used as a cross-linking agent to crosslink the GHSM sponge. The specimens were moved to 4 °C for 24 h, frozen at −20 °C for 24 h, −80 °C 24 h, and then lyophilized for 72 h.

### 2.4. Synthesis of Biphasic Scaffold and Study of Morphology

The biphasic scaffold used for osteochondral regeneration was synthesized by coupling the GHSM sponge (lower layer) and NGFK scaffold (upper layer) with 0.1% microbial transglutaminase. The samples were dehydrated by graded ethanol solutions (50% for 5 min, 75% for 5 min, 85% for 5 min, 95% for 5 min, and 100% for 10 min, repeated three times). A uniform thickness of gold was sputtered and coated on the surface of all samples, which were then observed under an electron microscope (Hitachi, Tokyo, Japan).

### 2.5. Identification of Crystal Phase and Functional Group

Powders were mounted onto a Ni filter of the sample holder, and an X-ray diffractometer (Rigaku Corporation, Tokyo, Japan) was scanned from 10° to 70° (at a speed of 2°/min, potential: 30 kV, current: 15 mA) to determine the structures of crystal under Cu KI (* = 0.15406 nm) radiation. The X-ray diffraction (XRD) patterns were analyzed using an auto-matching model in Jade 6.0 software (Materials Data, Livermore, CA, USA), with the direction database of the international center for diffraction data (ICDD).

Fourier transform infrared (FTIR) spectroscopy was used to analyze the functional groups of the different scaffolds by detecting the dipole moment change for the specific target molecule. FTIR spectra of the scaffolds were measured using a spectrophotometer (Perkin Elmer, Waltham, MA, USA) at a 450–4000 cm^−1^ wavelength range. Scaffold characterization was compared with natural bone as a control.

### 2.6. In Vitro Experiment

The Institutional Review Board of National Taiwan University Hospital (NTUH IRB no. 201704005 RINA) approved the experimental protocol for use of human mesenchymal stem cells (hMSCs). During total hip- and knee-joint arthroplasty surgery, hMSCs were collected from bone marrow aspirates and mononuclear cells were isolated by Ficoll-Paque PLUS (GE Healthcare, Amersham, UK) solution. All hMSCs used in the following experiments were from passages 3 to 4.

### 2.7. Cell Viability

The scaffold was added to high-glucose DMEM (Sigma-Aldrich) at a concentration of 0.2 g/mL, and the extract medium was prepared after incubating at 37 °C for 24 h. Following the ISO 10993-5 standard, a water-soluble tetrazolium (WST-1) assay was performed to evaluate the cell viability of L929 cells (BCRC Strain Collection, Hsinchu, Taiwan). A density of 5 × 10^3^ cells/well was seeded in 96-well plates and incubated at 37 °C for 1 d. The culture medium was removed and replaced with the prepared extract medium, and the samples and cells were incubated for 1 to 3 d. For the WST-1 assay, each well was pretreated with 10 µL reagent for 4 h. The amount of formazan formed during the incubation was determined by using a spectrophotometric reader (Sunrise ELISA plate reader, Tecan, Männedorf, Switzerland), with absorbance measured at 450 nm (reference filter at 600 nm). Cell viability was calculated using the following equation:

Cell viability (%) = [(OD experiment − OD background) × 100]/(OD control − OD background)(1)

### 2.8. Cytotoxicity

The CytoTox 96 assay kit (Promega, Madison, WI, USA), which measures extracellular lactate dehydrogenase (LDH), was used to assess the cytotoxicity effect. The suspension medium was transferred to new enzymatic assay plates and the LDH substrate solution was added. After a 30 min incubation, the stop solution was added, and the absorbance of each well at 490 nm was measured by using the ELISA reader (Sunrise ELISA plate reader, Tecan, Männedorf, Switzerland). Cytotoxicity was calculated using the following equation:

Cytotoxicity (%) = [(Experimental value − Negative control) × 100]/(Positive control − Negative control). (2)

### 2.9. Live/Dead Assay

To evaluate the live/dead status of cells within constructs, staining was performed using 4 μM calcein AM (Life Technologies, Thermo Fisher Scientific Inc., Carlsbad, CA, USA) and 4 μM propidium Iodide (PI; Life Technologies, Thermo Fisher Scientific Inc.) for 30 min. The live cells were identified by calcein AM, which emits green fluorescence upon excitation (~495 nm/~515 nm); while dead cells were stained by PI, which emits red fluorescence upon excitation (~540 nm/~615 nm).

### 2.10. Gene Expression

At predetermined time points, total RNA was extracted from the cell constructs using a Total RNA Miniprep Purification Kit (GeneMark, Zhubei, Taiwan). Random hexamers (Vivantis, CA, USA) were used to synthesize the first-strand cDNA. Real-time reverse transcription (RT-PCR) was performed using TOOLS 2X SYBR qPCR Mix (Biotools Co., Ltd., Taipei, Taiwan) and a CFX Connect Real-Time PCR Detection System (BioRad Laboratories, Hercules, CA, USA) with reverse transcriptase (Vivantis, CA, USA; Cat No: RTPL12) at the following parameters: denaturation (95 °C, 3 min), polymerase chain reaction (PCR) 40 cycles (95 °C, 20 s), annealing (60 °C, 30 s), and elongation (72 °C, 30 s). The relative gene expression of target genes was calculated by using the 2^−ΔΔCt^ method, with glyceraldehyde 3-phosphate dehydrogenase (GAPDH) used as an internal control. The relative fold-changes of five bone-related genes, including alkaline phosphatase (ALP), RUNX2, osterix (SP7), osteonectin (ON or SPARC), osteocalcin (OC or BGLAP), and COL1a1, were quantified at days 7, 14, 21, and 28 of the incubating cells with the scaffold. The relative fold-changes of four cartilage-related genes, i.e., cartilage oligomeric matrix protein (COMP), ACAN, COL2a1, and SOX9, were also quantified. The primer sequence used for osteogenic and chondrogenic differentiation are listed in Table 1, respectively.

### 2.11. Western Blotting

After four weeks of co-culture with GHSM or NGFK, three samples were selected from each group. Human MSCs from each group were washed and cleaved with pre-cooled lysis buffer (Biotools). Total protein concentration was determined with a BCA protein assay (Pierce Chemicals Co., Rockford, IL, USA). Equal amounts of protein were separated by sodium dodecyl sulfate-polyacrylamide gel electrophoresis (8–12% SDS-PAGE) and transferred to a PVDF membrane (MilliporeSigma, Burlington, MA, USA). The membranes were blocked with 5% (*w/v*) non-fat milk for 1 h at 25 °C. and then incubated with primary antibodies (Abcam, Cambridge, UK) against OC (ab13418, 1:1000), ON (ab14174, 1:1000), COL1A1 (ab229389, 1:1000), ACAN (ab3778, 1:1000), COL2A1 (ab34712, 1:1000), COMP (ab300555, 1:1000), and GAPDH (ab8245, 1:1000; as an internal control) overnight at 4 °C. The membranes were then incubated at 37 °C with secondary antibodies for 2 h. The proteins were visualized with enhanced chemiluminescence (ECL), and their relative intensity was normalized to the internal control and control group using ImageJ software (National Institutes of Health, Bethesda, MD, USA).

### 2.12. Biochemical Analysis

The content of sulfated glycosaminoglycan (sGAG) was determined using the 9-dimethyl methylene blue chloride (DMMB) method with a Blyscan assay kit (Biocolor, Carrickfergus, UK). The amounts of cell numbers were determined by extraction of total DNA using a genomic geno plus DNA extraction miniprep system (Viogen, Taipei, Taiwan) and the Quant-iT PicoGreen dsDNA assay kit (Thermo Fisher Scientific, Waltham, MA, USA). Bone- and cartilage-specific proteins were determined using a commercial ELISA kit (FineTest, Wuhan Fine Biotechnology, Wuhan, China), and the ratio of total sGAG to dsDNA was averaged across samples.

### 2.13. Immunofluorescence Staining

To perform immunofluorescence analysis, samples were incubated with primary antibodies from Abcam, including rabbit anti-ACAN monoclonal antibody (diluted at 1:200), rabbit anti-COL2A1 polyclonal antibody (diluted at 1:200), rabbit polyclonal anti-osteocalcin (diluted at 1:200), and rabbit monoclonal anti-COL1A1 (diluted at 1:200), overnight at 4 °C. The following day, the samples were washed and incubated with secondary antibodies (diluted at 1:200) for 30 min at room temperature. Nuclei were stained with 4′,6-diamidino-2-phenylindole (DAPI; Thermo Fisher Scientific). Finally, the samples were washed and examined by using a fluorescence microscope (Axio Imager ZI, Zeiss, Germany).

### 2.14. In Vivo Experiment

The animal experiments were conducted in accordance with the Guide for the Care and Use of Laboratory Animals. Adult male Wistar rats (weighing 190–210 g) were provided with standard chow and water ad libitum and housed in a temperature-controlled room (at 23 °C) with a fixed 12-h light/dark cycle. The Institutional Animal Care and Use Committee (IACUC) of the National Taiwan University, School of Medicine, Taipei, Taiwan (No. 20180364), pre-approved the protocol for animal experiments.

### 2.15. Generation of Osteochondral Defect

To generate an osteochondral defect, a transverse medial parapatellar incision was made, and the patella was laterally dislocated. A circular hole (1 × 1 mm) was drilled in both the medial and lateral femoral condyle until bleeding from the subchondral bone was observed. The scaffolds were then implanted into one of the defective sites. After three months of implantation, the joints were harvested for histological evaluation. Buffered 4% paraformaldehyde was used to fix the joints and the specimens were demineralized, dehydrated, defatted, cleared with xylene, and then embedded in wax.

### 2.16. Histological Staining

All specimens were fixed using formaldehyde and decalcified with Plank–Rychlo solutions. The specimens were then divided into anterior and posterior sections, embedded in paraffin, cross-sectioned and stained on glass slides with a thickness of 5 μm. Hematoxylin and eosin (H&E) and Alcian blue (AB)/periodic acid-Schiff (PAS; Polysciences Inc., Warrington, PA, USA) staining of rat articular sections was performed for histological analysis. Examination of the regeneration of bone and cartilage in the defects was observed under a light microscope (IX71; Olympus, Tokyo, Japan). Images were visualized and captured at 40× and 400× magnification.

### 2.17. Statistical Analysis

Quantitative data are presented as the mean ± standard deviation (SD). One-way ANOVA was used to assess the difference between groups and the level of statistical significance was set at *p* < 0.05. The statistical analysis was performed using SPSS Statistics 29 (IBM, Armonk, NY, USA).

## 3. Results

### 3.1. Characteristics of the Biomimetic Biphasic Scaffold (BPS)

#### 3.1.1. Morphology of the Biphasic Scaffold (BPS)

**Figure 1:**
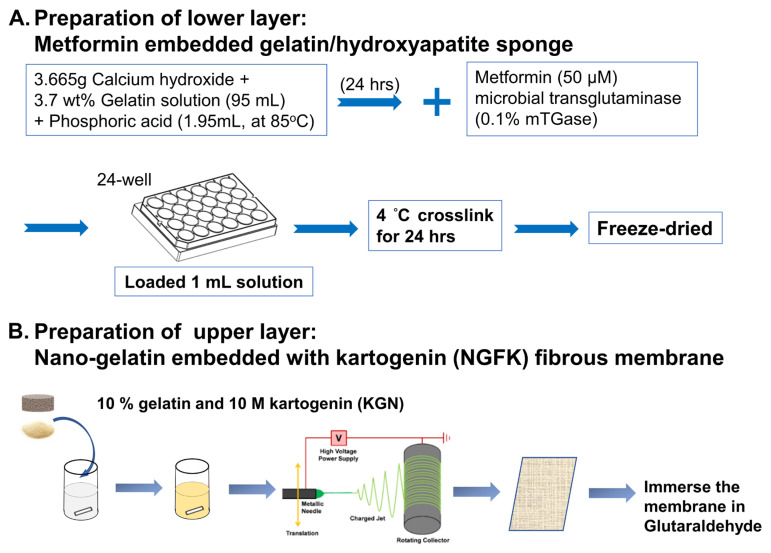
Synthesis of biphasic scaffold.

The surface morphology of the biphasic scaffold (BPS) was examined using scanning electron microscopy (SEM). The images showed that the mean pore size of the gelatin/hydroxyapatite scaffold embedded with metformin (GHSM) was 80.93 ± 16.7 µm (Figure 1), and the diameter of the nano-gelatin fiber embedded with kartogenin (NGFK) was 183 ± 57 nm (Figure 1). The biphasic scaffold (BPS) had a highly interconnected porous structure in the GHSM and multilayer structure in the NGFK, allowing distribution of optimal oxygen and nutrients throughout the scaffold.

#### 3.1.2. Crystal Phase Identification

X-ray diffraction (XRD) analysis of the GHSM showed a pattern similar to natural bone tissue (Figure 1). Broad diffractions corresponding to the (002), (211), (300), (202), (130), (002), (222), and (213) planes of the nature bone were observed in comparison to reference to the International Centre for Diffraction Data (ICDD). This analysis confirmed that hydroxyapatite (Hap) mineralization did occur in the gelatin/hydroxyapatite scaffold embedded with metformin (GHSM).

#### 3.1.3. Functional Group Identification

The infrared spectrum of the gelatin/hydroxyapatite scaffold embedded with metformin (GHSM) and nano-gelatin fiber embedded with kartogenin (NGFK) was analyzed to identify their functional groups (Figure 1). Peaks located in the 500–1100 cm^−1^ region were characteristic of hydroxyapatite (Hap), with the peak found at 1062 cm^−1^ corresponding to the asymmetric bending and stretching band of the (PO_4_)^3−^ group. The peaks observed at 1650 cm^−1^, 2800–2950 cm^−1^, and 3430 cm^−1^ were characteristic peaks for the C=O group, C-H stretching, and O-H stretching of gelatin, respectively. In the FTIR spectrum for the GHSM, characteristic bands were observed at 1625 cm^−1^ and 1569 cm^−1^ (corresponding to C-N stretching of MET), a band at 3150 cm^−1^ (due to N-H secondary stretching of MET), and two typical bands at 3290 cm^−1^ and 3370 cm^−1^ (relative to N-H primary stretching of MET).

### 3.2. In Vitro Study

#### 3.2.1. Biocompatibility of Biphasic Scaffold (BPS)

**Figure 2:**
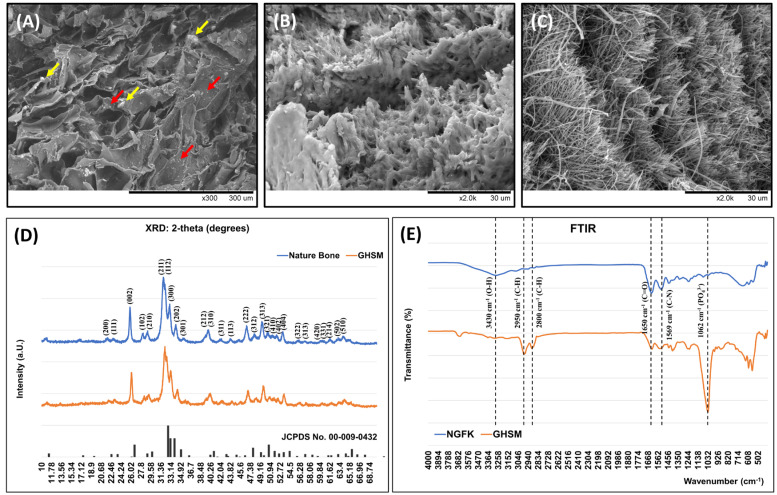
Characteristics of biphasic scaffold (BSP). Upper: SEM images of the (**A**) gelatin/hydroxyapatite (red arrows) scaffold embedded with metformin (yellow arrows) (GHSM), (**B**) interface of biphasic scaffold (BPS), and (**C**) nano-gelatin fiber embedded with kartogenin (NGFK). The BPS had an open and interconnected porous structure with a homogeneous pore distribution in both the gelatin/hydroxyapatite scaffold embedded with metformin (GHSM) and nano-gelatin fiber embedded with kartogenin (NGFK). The red arrow and yellow arrow indicated the Hap and MET, respectively. (**D**): XRD patterns of natural bone tissue, gelatin/hydroxyapatite scaffold embedded with metformin (GHSM), and reference JCPDS (Joint Committee on Powder Diffraction Standards) cards (No. 000-009-432). (**E**): The Fourier-transform infrared spectroscopy (FTIR) spectrum of the gelatin/hydroxyapatite scaffold embedded with metformin (GHSM) and nano-gelatin fiber embedded with kartogenin (NGFK).

To assess the biocompatibility of the biphasic scaffold (BPS), L929 fibroblasts were cultured with a scaffold, and the cell viability and cytotoxicity were evaluated using WST-1 and LDH assays on days 1, 2, and 3 of culturing (Figure 2). The viability of cells cultured with a biphasic scaffold (BPS) was similar to that of cells cultured under control conditions, indicating that the biphasic scaffold (BPS) is not toxic to L929 fibroblasts. Live/dead staining revealed no difference in the ratio of red (dead cells) to green signals (live cells) among the experimental and control groups, regardless of the concentration of the BPS extract. Therefore, we concluded that the biphasic scaffold (BPS) did not induce cytotoxicity.

#### 3.2.2. Osteo- and Chondrogenic Differentiation Potential of the Biomimetic Biphasic Scaffold

**Figure 3:**
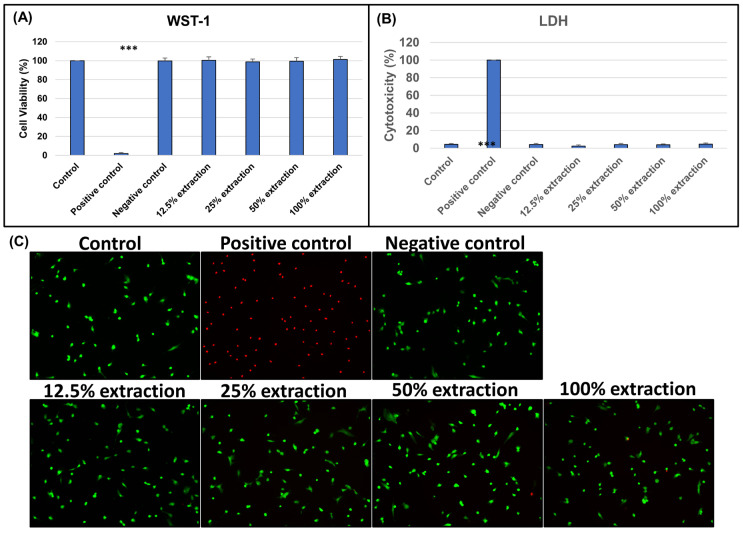
Evaluating the biocompatibility of biphasic scaffold (BPS). (**A**) Cell viability at different concentrations of biphasic scaffold (BPS) by WST-1 assay (n = 12, *** *p* < 0.001 compared with control). (**B**) Cytotoxicity at different concentrations of biphasic scaffold (BPS) by LDH assay (n = 12, *** *p* < 0.001 compared with control). (**C**) Live/dead staining at different concentrations of biphasic scaffold (BPS).

To evaluate the osteogenic differentiation potential of hMSCs on the gelatin/hydroxyapatite scaffold embedded with metformin (GHSM), we examined the expression of osteogenic genes using qPCR after 4 weeks of culture (Figure 3). The results showed a significant upregulation of *ALP*, *RUNX2*, *SP7*, *SPARC, BGLAP*, and *COL1A1* genes in hMSCs cultured on the GHSM, indicating their differentiation into pre-osteoblasts. To assess chondrogenic differentiation potential, we examined the expression of chondrogenic genes *COMP, ACAN, COL2A1*, and *SOX9* in hMSCs cultured on the nano-gelatin fiber embedded with kartogenin (NGFK) using qPCR (Figure 3). The results revealed a significant upregulation of these genes, indicating the differentiation of hMSCs into pre-chondroblasts.

Western blotting analysis confirmed the increased expression of osteogenic markers ON, OC, and COL1A1 and chondrogenic markers ACAN, COMP, and COL2A1 in hMSCs cultured on GHSM and NGFK, respectively (Figure 3). Moreover, the 1,9-dimethylmethylene blue (DMMB) assay showed a significant increase in the *sGAG/dsDNA* ratio over 4 weeks of culture on both the GHSM and NGFK (Figure 3), indicating chondrogenic differentiation and extracellular matrix production.

#### 3.2.3. Biochemical Analysis

**Figure 4:**
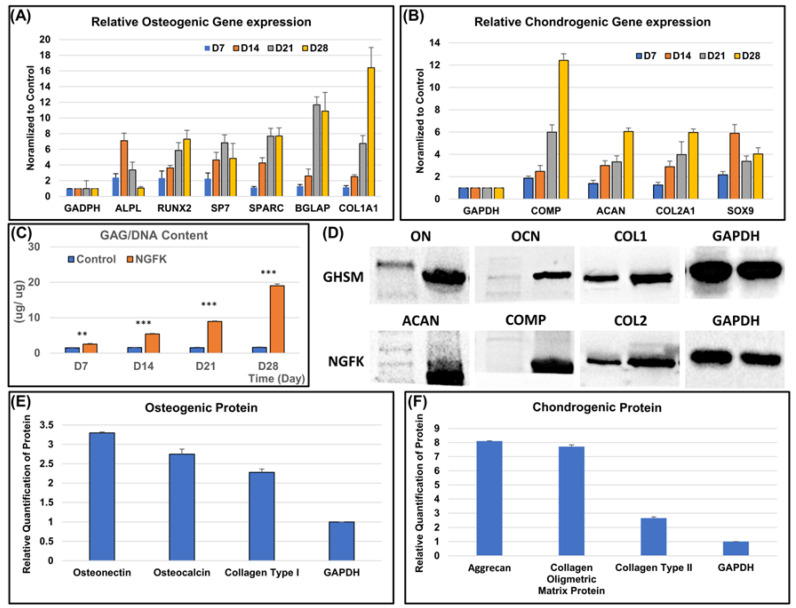
Osteo- and chondrogenic differentiation associated with the gelatin/hydroxyapatite scaffold embedded with metformin (GHSM) and nano-gelatin fiber embedded with kartogenin (NGFK). Relative osteogenic (**A**) and chondrogenic (**B**) gene expression in hMSCs cultured with the gelatin/hydroxyapatite/metformin scaffold loaded with metformin (GHSM) and electrospun scaffold loaded with kartogenin (NGFK), compared with the control group. Ratio of sGAG to dsDNA content (**C**) after 7, 14, 21, and 28 d of differentiation and Western blotting (**D**) of osteogenic and chondrogenic proteins of hMSCs co-cultured with the gelatin/hydroxyapatite/metformin scaffold loaded with metformin (GHSM) and electrospun scaffold loaded with kartogenin (NGFK) for 4 weeks. Quantitative analysis of osteogenic (**E**) and chondrogenic (**F**) protein expression. Amount of Western blotting product in hMSCs was taken as 1.0, and the relative ratio of the products is indicated in the ordinate. The value was corrected using the expression of GAPDH as an internal control. (**: *p* < 0.01, ***: *p* < 0.001).

**Figure 5:**
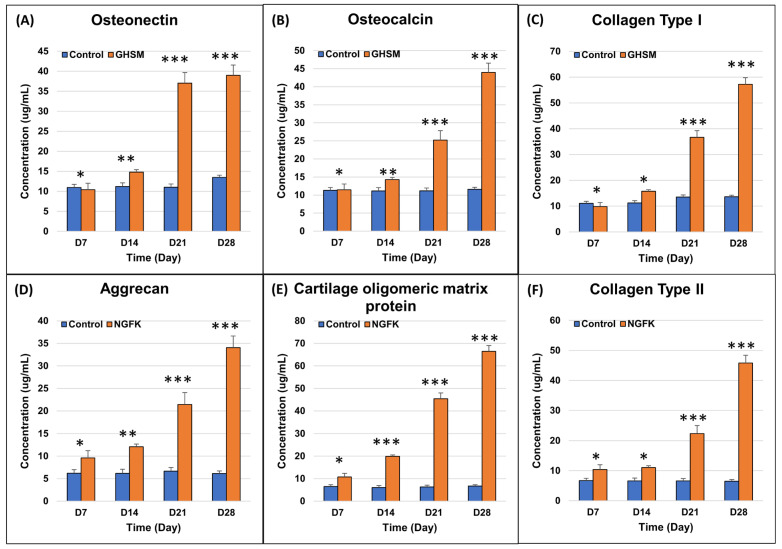
Osteo- (**A**–**C**) and chondro- (**D**–**F**) specific biomarker expression for the gelatin/hydroxyapatite scaffold embedded with metformin (GHSM) and nano-gelatin fiber embedded with kartogenin (NGFK) co-cultures, respectively. n = 12, * *p* < 0.05, ** *p* < 0.01; *** *p* < 0.001.

ELISA analysis revealed that the expression levels of ON, OC, and COL1A1 were significantly upregulated compared to the control group and were also increased with the increasing culture time (Figure 4 and Figure 5). Similarly, the expression levels of ACAN, COMP, and COL2A1 were upregulated when compared to the control group.

#### 3.2.4. Immunofluorescence Analysis

**Figure 6:**
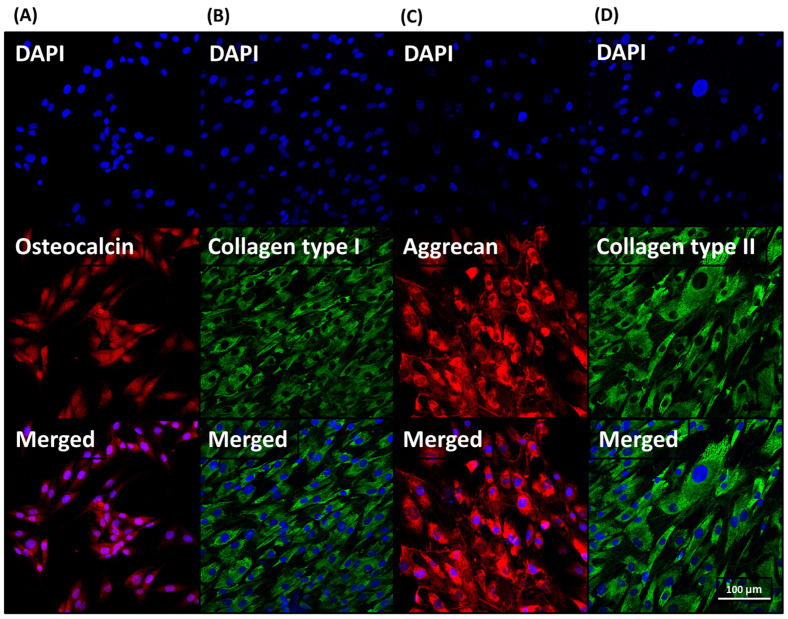
Immunofluorescence staining of osteo- (**A**,**B**) and chondro- (**C**,**D**) specific biomarkers. Osteocalcin and aggrecan were stained in red, and the different types of collagen were stained in green.

After 4 weeks of culture with the gelatin/hydroxyapatite scaffold embedded with metformin (GHSM) and nano-gelatin fiber embedded with kartogenin (NGFK), hMSCs were subjected to immunofluorescence staining. On day 28, positive staining for osteogenic and chondrogenic proteins was observed (Figure 6). These results suggest that the biphasic scaffold (BPS) facilitates the attachment of hMSCs and the production of an osteochondral tissue construct.

### 3.3. In Vivo Histological Evaluation

**Figure 7:**
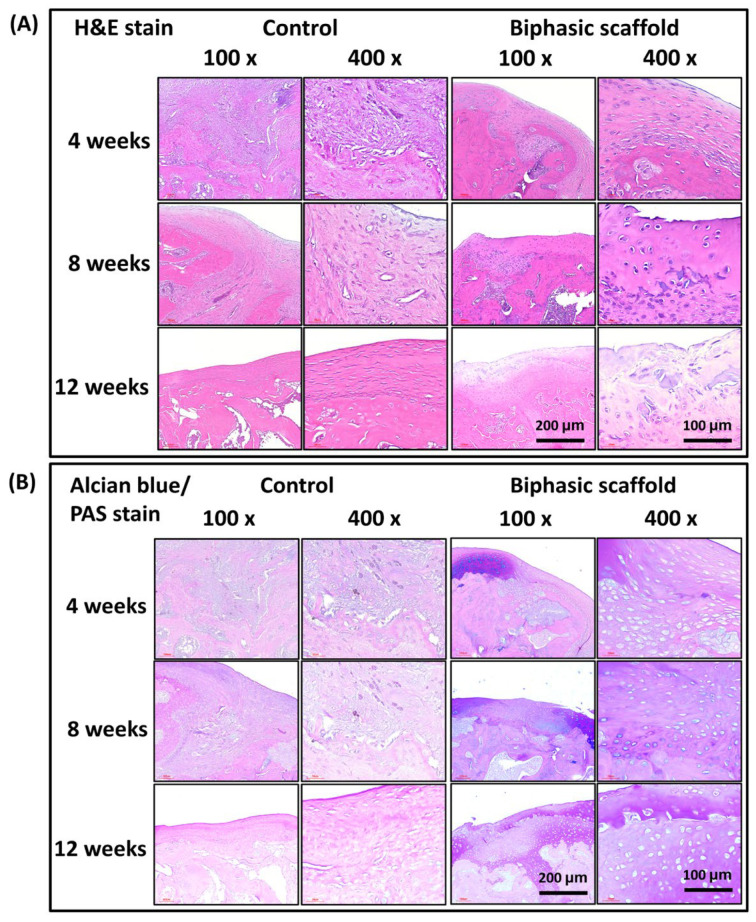
Histological staining. (**A**) Hematoxylin and eosin (H&E) staining of subchondral bone. (**B**) Alcian blue and periodic acid–Schiff (AB/PAS) double staining of cartilage regeneration. The positive reaction between AB and PAS makes neutral and acidic mucosubstances appear purple.

After 12 weeks of post-implantation, the formation of osteochondral tissue in defective rat joints was evaluated by histological staining in the control and BPS groups. In the experimental groups, new bone regeneration at the defect site was confirmed by hematoxylin and eosin (H&E) staining (Figure 7). On the other hand, the control group showed incomplete trabecular bone formation, with fibrous connective tissue filling most of the defect sites. The sections from the experimental group, after BPS implantation, showed positive double staining with Alcian blue (AB) and Periodic acid–Schiff (PAS) stains (Figure 7). The positive AB staining indicated glycosaminoglycans (GAGs) accumulation, imparting a blue color to acidic mucins and other carbonylated or weakly sulfated acid muco-substances. This finding suggests that the healing of articular cartilage and subchondral bone was significantly better in the biphasic scaffold (BPS) group than those in the control group.

## 4. Discussion

The osteochondral unit experiences high pressure, frequent motion, and complex interactions with the subchondral bone, which are further compounded by the limited healing potential of articular cartilage [28]. Conservative management is the current treatment for articular defects or osteoarthritis, but no single solution has demonstrated complete and durable functional repair of osteochondral lesions [29]. The development of improved and innovative therapeutic approaches to promote osteochondral tissue regeneration is imperative due to the immense socioeconomic concern of osteochondral-related problems without suitable long-term treatment options [30]. Because cells from different sources may have different differentiation potentials, the selection of appropriate cell sources for osteochondral tissue engineering is a critical issue [31]. Although osteochondral tissue can be engineered from various cell types, mesenchymal stem cells (MSCs) are still the preferred cell type [32].

Electrospinning has emerged as a promising approach in osteochondral tissue engineering due to its ability to provide mechanical properties to the scaffolds while supporting cell growth [33]. By mimicking the fibrils embedded in the extracellular matrix of natural tissues, fibrous structures in scaffolds can replicate the morphology and scale of native tissue. The use of microfibers and/or nanofibers in scaffolds has shown promise in enhancing cell recruitment, infiltration, and differentiation, even in the absence of growth factors [34]. The small diameter of fibers produced by electrospinning provides a higher surface area to volume ratio, better tunable porosity, and enables the integration of more bioactive molecules to enhance cellular response [35].

In addition to growth factors, small molecules have also emerged as potential modulators of MSC behavior. These molecules are cost-effective and can selectively alter stem cell behavior during differentiation, making them attractive options for clinical use. In this study, we aimed to promote regeneration of osteochondral defects using the small molecules kartogenin (KGN) and metformin (MET) embedded in a biphasic scaffold (BPS). The BPS is composed of a bone layer scaffold, gelatin/hydroxyapatite scaffold embedded with metformin (GHSM), and a cartilage layer, nano-gelatin fiber embedded with kartogenin (NGFK).

Johnson’s first report showed that kartogenin (KGN) could induce in vitro differentiation of human mesenchymal stem cells (hMSCs) into hyaline cartilage nodules containing proteoglycans and collagen II [36]. In osteoarthritis (OA), KGN can prevent cartilage degeneration and subchondral bone changes [37]. Furthermore, KGN-incorporated scaffolds have shown excellent effects on cartilage regeneration by recruiting endogenous cells from the host without the need for cell transplantation [38]. KGN selectively upregulates the expression of chondrogenic markers such as ACAN, COL2A1, SOX9, and lubricin to promote chondrogenic differentiation [39]. Our study found similar results in our biphasic scaffold.

Metformin (MET) exerts direct osteogenic effects by activating AMP-activated protein kinase (AMPK) and promoting osteoblast differentiation, which can antagonize bone loss, maintain bone mineral density, and protect bone microarchitecture [40]. Metformin’s osteogenic potential is attributed to the upregulation of RUNX2 expression via the AMPK signaling pathway in MSCs, pre-osteoblasts, and osteoblasts [41,42]. In vitro, metformin can upregulate Runx2 expression in bone marrow mesenchymal cells, increase their ALP activity, collagen synthesis, osteocalcin production, and extracellular calcium deposition [43]. Runx2 phosphorylation is critical for osteogenesis as it can stimulate the secretion of bone sialoprotein (BSP) and differentiation MSCs or pre-osteoblasts to osteoblasts [44]. Conversely, the loss of Runx2 may activate adipogenesis, which is directly related to the AMPK-mediated phosphorylation of Runx2 serine [45]. In vivo, metformin can reduce bone loss by inducing osteoblast genes, such as Lrp5 and Runx2 [46], stimulate osteoprotegerin (OPG) expression, and reduce receptor activator RANKL levels [47]. Our study also demonstrated similar osteogenic effects in vitro.

To ensure clinical safety, autologous human serum is preferable to fetal bovine serum (FBS) for use in musculoskeletal tissue engineering. The most favorable cell source for cartilage regeneration are endogenous mesenchymal stem cells (MSCs). In this study, a novel biphasic scaffold (BPS) showed promising results in enhancing both subchondral bone repair and cartilage regeneration. In vitro, MSCs adhered, proliferated, and differentiated on the gelatin/hydroxyapatite scaffold embedded with metformin (GHSM) and nano-gelatin fiber embedded with kartogenin (NGFK) layers. In vivo, after implantation of the biphasic scaffold (BPS) [which contains both kartogenin (KGN) and metformin (MET)] into the sites of osteochondral defects without exogenous cell seeding, the biphasic scaffold (BPS) promoted the complete regeneration of osteochondral defects in rats. This study effectively enabled the recruitment of endogenous bone marrow MSCs for osteogenesis and chondrogenesis, facilitating osteochondral regeneration. However, the in vitro drug release and degradation profile was not evaluated in this study and the relative time-related osteogenic and chondrogenic gene expression effects were not analyzed; the possible time-related benefit from different components may have been overlooked. In this study, we used an empty osteochondral defect (without control scaffold) as a control; another sham control with a control scaffold can be used to validate the role of small molecules in osteochondral regeneration. Although there have been great advances in musculoskeletal tissue engineering, current clinical treatments still have drawbacks in reproducing hyaline cartilage with a full function [48]. The gene-activated cell-free strategy offers a better scaffold for osteochondral reconstruction. In this study, the metformin/kartogenin-based cell-free osteochondral scaffold has been demonstrated to completely restore osteochondral defects. By developing an electrospun biphasic scaffold capable of mimicking the extracellular matrix, better tissue maturation and clinical outcomes can be achieved.

## 5. Conclusions

In conclusion, the composite biomimetic biphasic scaffold (BPS) demonstrated excellent performance in repairing osteochondral defects in rats. This scaffold, based on small-molecule drugs and an extra-cellular matrix (ECM), provides a micro-physiological joint organoid model system for studying spatiotemporal responses across the osteochondral interface. By using small molecules, this study not only demonstrated in vitro expansion and differentiation of endogenous stem cells but also direct in vivo modulation of stem cells. The biphasic osteochondral scaffold was spatially fabricated with biochemical cues to guide specific functional tissue regeneration. Site-specific delivery of inducible and tunable gene signals provides spatial control over both the phase of osteochondral composition and the remodeling. The biphasic scaffold (BPS) showed excellent biocompatibility and enabled rapid osteo- and chondro-neogenesis, further supporting its potential for clinical application. Based on our findings, the regeneration of osteochondral defects may be more feasible when small molecules rather than growth factors are used. Despite the promising results of this study, small animal models may not be the most suitable models for assessing clinical outcomes, and investigating the biphasic scaffold (BPS) in large animal studies is imperative in the near future.

## Figures and Tables

**Table 1:** Primers used for osteogenic and chondrogenic differentiation.

Osteogenic Gene		
Gene Name	Forward Primer (5′–3′)	Reverse Primer (5′–3′)
ALP	GAGAAGCCGGGACACAGTTC	CCTCCTCAACTGGGATGATGC
RUNX2	TAGGCGCATTTCAGGTGCTT	GGTGTGGTAGTGAGTGGTGG
SP7	TAGGACTGTAGGACCGGAGC	CATAGTGAACTTCCTCCTGGGG
SPARC	ATTGACGGGTACCTCTCCCA	GAAAAAGCGGGTGGTGCAAT
BGLAP	CTCACACTCCTCGCCCTATTG	GCTTGGACACAAAGGCTGCAC
COL1A1	AGAGGTCGCCCTGGAGC	CAGGAACACCCTGTTCACCA
GAPDH	AATGGGCAGCCGTTAGGAAA	GCCCAATACGACCAAATCAGAG
**Chondrogenic Gene**		
**Gene Name**	**Forward Primer (5′–3′)**	**Reverse Primer (5′–3′)**
SOX9	GGCAAGCTCTGGAGACTTCTG	CCCGTTCTTCACCGACTTCC
ACAN	TAAAAAGGGCACAGCCACCAC	GTGAGCTCCGCTTCTGTAGTC
COMP	CAAGGCCAACAAGCAGGTTT	TATGTTGCCCGGTCTCACAC
COL2A1	ATGAGGGCGCGGTAGAGA	GCCAGCCTCCTGGACATC
GAPDH	AATGGGCAGCCGTTAGGAAA	GCCCAATACGACCAAATCAGAG

Note: ALP: Alkaline phosphatase; RUNX2: Runt-related transcription factor; SP7: Osterix; ON or SPARC: Osteonectin; OC or BGLAP: Osteocalcin; COL1A1: Collagen type 1A1; GAPDH: glyceraldehyde 3-phosphate dehydrogenase; SOX9: SRY (sex determining region Y)-box 9; ACAN: Aggrecan; COMP: Cartilage oligomeric matrix protein; COL2A1: Collagen type 2A1; GAPDH: glyceraldehyde 3-phosphate dehydrogenase.

## Data Availability

The datasets used and/or analyzed in the current study are available from the corresponding author on reasonable request.

## Supplementary Materials

The following supporting information can be downloaded at: https://www.mdpi.com/article/10.3390/bioengineering10070847/s1, Figure S1: Flow chart of experimental design.
