# Supplementary figures and images for: Small-Molecule Loaded Biomimetic Biphasic Scaffold for Osteochondral Regeneration: An In Vitro and In Vivo Study
Source: Bioengineering (Basel). 2023 Jul 17;10(7):847. doi: 10.3390/bioengineering10070847 (PMC10376318; doi:10.3390/bioengineering10070847)

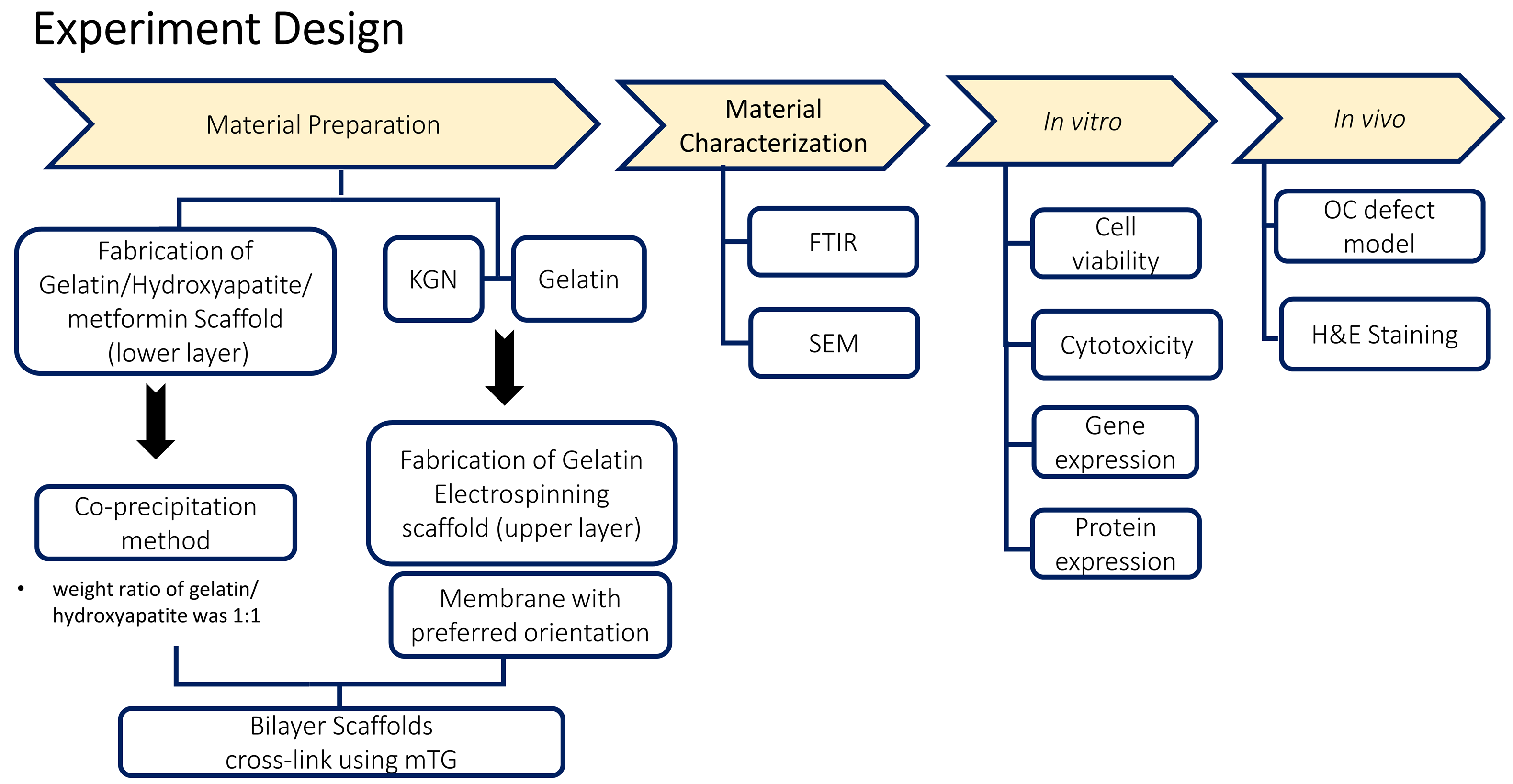

Supplement: Supplementary file 1 [file bioengineering-10-00847-s001.zip › bioengineering-2415695-supplementary.tif]
